# Causal association of epigenetic aging and COVID-19 severity and susceptibility: A bidirectional Mendelian randomization study

**DOI:** 10.3389/fmed.2022.989950

**Published:** 2022-09-23

**Authors:** Wenchang Xu, Fengjun Zhang, Yingzhou Shi, Yuanzhen Chen, Bin Shi, Gongchang Yu

**Affiliations:** ^1^School of Acupuncture and Tuina, Shandong University of Traditional Chinese Medicine, Jinan, China; ^2^Neck-Shoulder and Lumbocrural Pain Hospital of Shandong First Medical University, Shandong First Medical University and Shandong Academy of Medical Sciences, Jinan, China; ^3^Shandong Provincial Hospital, Shandong University, Jinan, China

**Keywords:** Mendelian randomization, aging, COVID-19, epigenetic age, telomere length, genome-wide association study (GWAS), SARS-CoV-2

## Abstract

Observational data from China, the United States, France, and Italy suggest that chronological age is an adverse COVID-19 outcome risk factor, with older patients having a higher severity and mortality rate than younger patients. Most studies have gotten the same view. However, the role of aging in COVID-19 adverse effects is unclear. To more accurately assess the effect of aging on adverse COVID-19, we conducted this bidirectional Mendelian randomization (MR) study. Epigenetic clocks and telomere length were used as biological indicators of aging. Data on epigenetic age (PhenoAge, GrimAge, Intrinsic HorvathAge, and HannumAge) were derived from an analysis of biological aging based on genome-wide association studies (GWAS) data. The telomere length data are derived from GWAS and the susceptibility and severity data are derived from the COVID-19 Host Genetics Initiative (HGI). Firstly, epigenetic age and telomere length were used as exposures, and following a screen for appropriate instrumental variables, we used random-effects inverse variance weighting (IVW) for the main analysis, and combined it with other analysis methods (e.g., MR Egger, Weighted median, simple mode, Weighted mode) and multiple sensitivity analysis (heterogeneity analysis, horizontal multiplicity analysis, “leave-one-out” analysis). For reducing false-positive rates, Bonferroni corrected significance thresholds were used. A reverse Mendelian randomization analysis was subsequently performed with COVID-19 susceptibility and severity as the exposure. The results of the MR analysis showed no significant differences in susceptibility to aging and COVID-19. It might suggest that aging is not a risk factor for COVID-19 infection (*P*-values are in the range of 0.05–0.94). According to the results of our analysis, we found that aging was not a risk factor for the increased severity of COVID-19 (*P* > 0.05). However, severe COVID-19 can cause telomere lengths to become shorter (beta = −0.01; se = 0.01; *P* = 0.02779). In addition to this, severe COVID-19 infection can slow the acceleration of the epigenetic clock “GrimAge” (beta = −0.24, se = 0.07, *P* = 0.00122), which may be related to the closely correlation of rs35081325 and COVID-19 severity. Our study provides partial evidence for the causal effects of aging on the susceptibility and severity of COVID-19.

## Introduction

SARS-CoV-2 caused the Coronavirus Disease 2019 (COVID-19) pandemic, which has evolved into a major global health threat. Globally, more than 517 million COVID-19 cases were confirmed by May 2022. According to the World Health Organization (1), the COVID-19 death toll has reached 6.26 million worldwide. Thus, to protect high-risk groups against COVID-19, risk factors associated with increased susceptibility to the disease must be identified (2). In COVID-19, age is an important risk factor, and the older you get, the more severe and mortality you become (3–6). Italy’s COVID-19 mortality data (CFR) shows that age has a significant impact (7), with case fatality rates ranging from less than 0.4% or less in patients in their 40 s, 1% in 50 s, 3.5% in 60 s, 12.8% in 70 s, and 20.2% in 80 s and older; the total case fatality rate was 7.2%. Other countries such as China (8), the United States (9), and France (10) have achieved similar results, with older COVID-19 case fatality rates being higher than among younger patients. Not only that, but the data tested in Italy show that the total case fatality rate in Italy is higher than that in China (7.2% and 2.3%, respectively), which may be related to the fact that the proportion of elderly people in Italy is higher than that in China.

Aging-related biological processes are reflected in molecular hallmarks such as epigenetic modifications and telomere attrition (11–13). Epigenetic age has recently emerged as a promising indicator of cellular senescence and may be more strongly correlated with mortality than earlier indicators of biological age (14). Different from chronological age, epigenetic age is a heritable indicator of biological aging derived from DNA methylation (DNAm) data. Each indicator (epigenetic clock) is based on the unique characteristics of DNAm levels reflecting biological aging, as measured at a specific set of cytosine-phosphate-guanine (CpG) loci (15). “First-generation” epigenetic clocks such as HannumAge (16) and Intrinsic HorvathAge (17) are calculated from DNAm levels at CpG loci that are closely associated with chronological age, and better predict chronological age than other clocks. There are 71 age-related CpG in the blood, and HannumAge results from training on these loci. The HorvathAge is trained on the 353 age-related CpG species found in human tissues and cells, and then further adjusts for the blood cell count. “Second-generation” epigenetic clocks, like PhenoAge (18) and GrimAge (19), integrate data from nine clinical biomarkers (e. g., white blood cell count, C-reactive protein, lymphocyte percentage, albumin, creatinine, etc.) and 513 CpG associated with mortality. The CpG component site of GrimAge is a surrogate for disease-related and health-related proteins and smoking history, it shows a high association with all-cause mortality and age-related health conditions and has a good ability to predict both morbidity rates and mortality (20). SARS-CoV-2 has been discovered to induce changes in DNA methylation, which affects the expression of immune response suppression genes. Studies have shown that severe COVID-19 infection accelerates epigenetic age aging, but this was not absolute, and epigenetic age reversal occurred in the later stages of infection (21). Besides, leukocyte telomere depletion, another hallmark of aging, is associated with increased human lifespan and risk of age-related diseases (22–24), leading to the development of DNA age reversal-based telomere length estimators (25). Most mammals lack the ability to fully replicate the ends of linear DNA molecules when cells proliferation expressing telomerase, and telomere-protective sequences at chromosome ends are gradually consumed and lost with DNA replication. This feature makes telomere length one of the indicators that can predict biological age. The epigenetic clock and telomere length measure the different characteristics of biological aging, and the Marioni studies showed no correlation between telomere length and epigenetic age (26).

Epigenetic age is greater than chronological age in various disease contexts and lowers in long-lived humans, providing strong evidence that epigenetic age reflects biological age. In short, epigenetic age can reflect chronological age but they are not identical. Although lots of studies have shown that chronological age is related to the severity of COVID, no studies have been able to prove a causal link between epigenetic age and COVID -19. In this study, we used the MR analysis approach to account for the causal relationship between COVID-19 and aging, whereas the MR study used genetic variants that are reliably associated with modifiable risk factors. Therefore, confusion can be minimized and reverse causality ruled out since variation is randomly assigned from parent to offspring at conception.

## Research design and methods

### Research design

**FIGURE 1:**
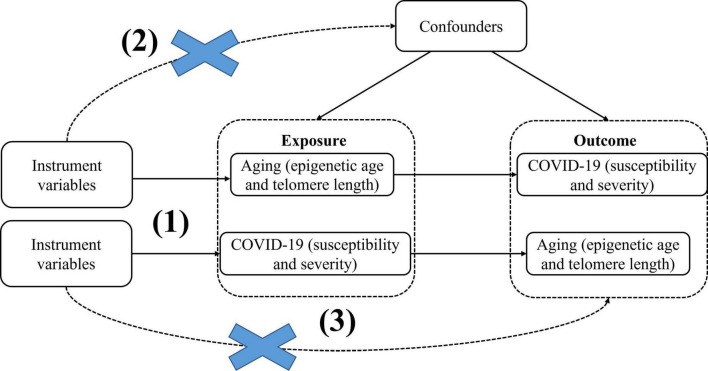
BidirectionalMendelian randomization paradigm and assumptions of aging and COVID-19. Mendelian randomization assumptions: (1)the instrument variants must be closely related to the exposure, (2) the instrument variants must be independent of any confounder of the exposure-outcome association, (3) the instrument variants must be associated with the outcome only *via*the exposure.

Here, we use epigenetic age (PhenoAge, GrimAge, Hannum, HorvathAge) and telomere length as “exposure,” respectively, and COVID-19 severity and susceptibility as “outcome,” to screen out the instrumental variables for bidirectional Mendelian randomization analysis, significance thresholds were corrected using Bonferroni to reduce the probability of false positives and assess heterogeneity using the Cochran Q analysis, and finally perform sensitivity analysis (Horizontal pleiotropy analysis and “leave-one-out” analysis) to verify the reliability of causal results. We then performed reverse MR with COVID-19 severity and susceptibility as “exposure” and epigenetic age and telomere length as “outcomes.” MR studies need to meet the following three key assumptions: (1) Instrumental variables must be closely associated with exposure factors; (2) The instrumental variables are not be associated with any confounding factors associated with exposure factors and outcome factors; (3) Instrumental variables do not affect the results unless they may be associated with exposure (Figure 1). In this study, bidirectional Mendelian randomization was used to evaluate the causal connection between age aging and susceptibility and severity of COVID-19.

### Data sources

Recent genome-wide association studies based on a meta-analysis of European ancestry of 34,710 participants from 28 cohorts, identify 137 loci for DNA biomarkers related to aging^1^ (Table 1). From this study, we obtained summary genetic association estimates for epigenetic age acceleration measures of HannumAge (16), Intrinsic HorvathAge (17), PhenoAge (18), and GrimAge (19). The analysis included 28 European ancestry studies with 57.3% female participants. For more information and a detailed description of the methods, see the latest meta-analysis of biological aging by GWAS (27).

**TABLE 1:** Description of contributing studies.

Type	Phenotype	Population	SNP	Simple size	n_cases	n_controls	Release Date	Access address	DOI
COVID-19 severity	Very severe respiratory confirmed COVID vs. population	Europeans	4225986	1388342	5101	1383241	18-Jan-21	https://www.covid19hg.org/results/r5/	https://doi.org/10.1038/s41431-020-0636-6
COVID-19 susceptibility	COVID vs. population	Europeans	7090490	1683768	38984	1644784	18-Jan-21	https://www.covid19hg.org/results/r5/	https://doi.org/10.1038/s41431-020-0636-6
Epigenetic age	PhenoAge GrimAge Hannum HorvathAge	Europeans	phenoAge(7567585) GrimAge(7567701) Hannum(7565045) HorvathAge(7567532)	34710	–	–	1-Jul-20	https://datashare.ed.ac.uk/handle/10283/3645	https://doi.org/10.7488/ds/2834
Telomere length	telomere length	Europeans	20134421	472174	–	–	24-Mar-21	https://gwas.mrcieu.ac.uk/datasets/ieu-b-4879/	https://doi.org/10.1101/2021.03.23.21253516

We obtained the genetic associations of COVID-19 susceptibility and severity from the 5th round of the COVID19-hg GWAS meta-analysis, released on January 18,2021, which provides publicly accessible summary statistics available about several COVID-19 outcomes from different studies that are publicly accessible (e.g., United Kingdom Biobank, FinnGen) (Table 1) (28). Documentation on the COVID-19 Host Genetics Initiative identified COVID-19 susceptibility and severity phenotypes as C2 (COVID-19 patients vs. population which were defined as any individuals who never had COVID-19, which included 38,984 COVID-19-positive cases and 1,644,784 controls) and A2 (hospitalized individuals with COVID-19 who died or required respiratory support vs. individuals without severe COVID-19 including those without COVID-19, which included 5,101 COVID-19 patients with severe symptoms and 1,383,241 controls). Support for the respiratory system is characterized by intubation-ventilator-assisted breathing or high-flow nasal cannulas. However, neither the age nor sex of this cohort was reported (Table 1). Further new releases and information can be obtained from the COVID-19 HGI homepage: https://www.covid19hg.org/results/r5/.

Genome-wide association study data for telomere length are obtained by https://gwas.mrcieu.ac.uk/. 472174 gender-insensitive Europeans were used as data sources. GWAS ID: ieu-b-4879, obtained from the uk/datasets website, whose brief information is shown in Table 1.

### Selection of instrumental variables

Referring to the whole genome, SNPs with significant (*P* < 5.0 × 10^–8^), set the parameter r^2^ threshold of 0.001 and kilobase pair (kb) to 10,000, and the LD_clumping function were pooled to exclude interference in linkage disequilibrium. Missing SNPs from the outcome database were removed. Analysis was performed using the R software package TwoSampleMR V.4.0 (29, 30). Finally, valid SNPs significantly associated with exposure were obtained as instrumental variables (IV). If the correlation between IV and exposure factors is weak, it is prone to a weak instrumental variable bias. To avoid weak instrumental variable bias, F values were calculated in this study. The F value is the ratio of the variance to the residual variance explained by the first stage model of Mendelian randomization. It is generally assumed that there is no weak instrumental variable bias (31) at *F* > 10. The proportion of trait variance explained by genetic tools (R^2^) and tool strength (F-statistics) was calculated using *R*^2^ =(2*MAF*(1−*MAF*)*β2)/(SE^2^*N) and *F* = *R*^2^(*N*−*k*−1)/(k*(−1−*R*^2^)) (MAF = minor allele frequency, β = effect size, SE = standard error, N = sample size, k = number of instrumental variables) (32). Finally, data were extracted from the outcome database and collated and merged in order to correspond the same effect allele with the effect values of exposure and outcome.

### Statistical analysis

The effect of exposure on outcome was analyzed by Wald ratio for each SNP. The effect of each SNP is given at the normalized log-transformed exposure level. The primary MR analysis was performed using the inverse variance weighted (IVW) method, where the SNP to outcome estimate is regressed on the SNP to exposure estimate. Then we calculated the causal effect estimates (equivalent to beta coefficients) and converted them to odds ratios (OR). This approach will provide the highest statistical power if three of MR’s key assumptions (described in the research design) are satisfied. Considering different patterns of violations, we performed a series of sensitivity analyses to assess and validate these assumptions.

First, we used the MR–Egger method, which allowed for an additional intercept (alpha) term that also provided an estimate of directional horizontal pleiotropy. This method relied upon the assumption that the size of a genetic variant’s direct effect on the outcome that did not operate through the exposure is independent of the variant’s effect on the exposure. In addition, we used four other meta-analysis methods that were known to be more reliable for the existence of horizontal pleiotropy: weighted median, simple mode, penalized weighted median, and weighted mode (33). We calculated the global Q-statistics and analyzed the MR-Egger intercept term to assess substantial heterogeneity and directional pleiotropy. In addition, we performed “leave-one-out” analyses for each SNP to examine whether there were high-impact instrumental variables that might have a disproportionate impact on MR results. Analyses were performed using primarily the TwoSampleMR package of the statistical software R (version: 4.0.0).

## Results

### Instrumental variable

Epigenetic age (numbers of instruments: PhenoAge = 11, R^2^ = 0.44%, F-statistic = 152.2; GrimAge = 4, R^2^ = 0.20%, F-statistic = 70.3; Hannum = 9, R^2^ = 1.13%, F-statistic = 155.4 and HorvathAge = 24, R^2^ = 1.26%, F-statistic = 442.4; total study populations = 34,710 cases) (Supplementary Table 1) and telomere length (numbers of instruments = 154, R^2^ = 1.18%, F-statistic = 36.63) had sufficient genome-wide loci (≥ 2) for MR analyses (Supplementary Table 2). Conversely, two COVID-19 phenotypes (numbers of instruments: severity = 4, R^2^ = 0.0037%, F-statistic = 51.2; susceptibility = 6, R^2^ = 0.0056%, F-statistic = 94.5; total study populations: severity = 1,388,342; susceptibility = 1683768) had sufficient genome-wide loci for reverse MR analyses (Supplementary Table 3). In Mendelian randomization, the index to evaluate the strength of an instrument variable is the F-statistic, and when the F value is greater than 10, it is a strong instrumental variable (34). In our analysis, the instrument strength was strong (F-statistic in bidirectional MR analyses range from 51.2 to 442.4), so we found no evidence of weak instrumental variable bias. Therefore, these instrumental variables are good estimates of the causal effect of exposure on the outcome.

### The Mendelian randomization results did not reveal a causal relationship between epigenetic age and COVID-19 susceptibility

**FIGURE 2:**
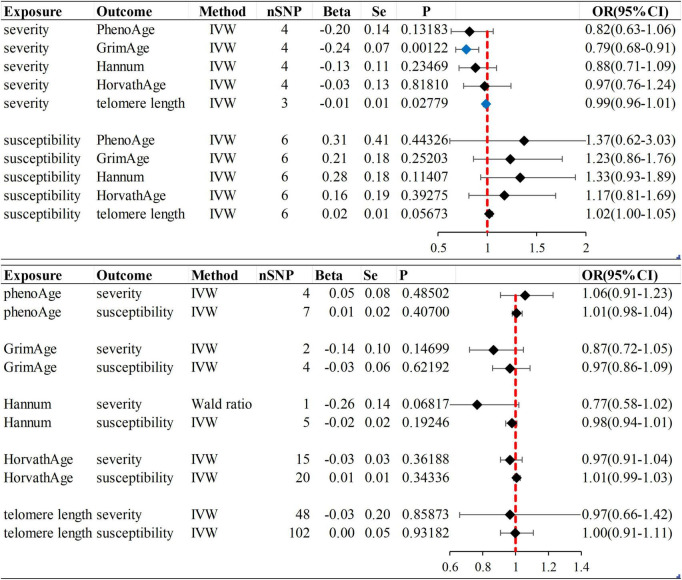
Inverse variance weighted (IVW) method was used as the main method to analyze the causal association between aging and susceptibility and severity of COVID-19. Aging is based on epigenetic age (PhenoAge, GrimAge, Hannum, HorvathAge) and telomere length as biological indicators. Beta: risk index; Se: standard error; OR (95% CI): odds ratio (95% confidence interval); Forest plot: Visualize the causal effect of exposure on the risk of outcome by IVW method [The standard line is the line of “X = 1” (red dashed line)], The blue marker dot is a positive result of *P* < 0.05).

We observed no causal relationship between epigenetic age and COVID-19 susceptibility from Mendelian randomization. The OR value and 95%CI calculated by inverse variance weighting are phenoAge and susceptibility: 1.01 (95%CI 0.98–1.04), *P* = 0.41; GrimAge and susceptibility: 0.97 (0.86–1.09), *P* = 0.62; Hannum and susceptibility: 0.98 (0.94–1.01), *P* = 0.19 and HorvathAge and susceptibility: 1.01 (0.99–1.03), *P* = 0.34. That is, there is no statistical significance (Figure 2) (Supplementary Figure 1). Mendelian randomization effect forest plot for a single SNP (Supplementary Figure 2). In reverse MR (exposure: COVID-19 susceptibility, outcome: epigenetic age), no significantly different values were also found (susceptibility and PhenoAge: beta = 0.31, se = 0.41, *P* = 0.44; susceptibility and GrimAge: beta = 0.21, se = 0.18, *P* = 0.25; susceptibility and Hannum: beta = 0.28, se = 0.18, *P* = 0.11 and susceptibility and HorvathAge: beta = 0.16, se = 0.19, *P* = 0.39). The results of the other analysis methods are visible in Supplementary Figure 3. Mendelian randomization effect forest plot for a single SNP (Supplementary Figure 4). Then we performed heterogeneity analysis and intercept term analysis of MR-Egger regression, with no significant heterogeneity and pleiotropy. The results are shown in Table 2.

**TABLE 2:** Heterogeneity and pleiotropic analysis for epigenetic age and susceptibility and severity of COVID-19.

		Q-statistics		Pleiotropic test	
Exposure	Outcome	MR Egger	IVW	egger_intercept	pval
severity	PhenoAge	Q = 3.732769 P = 0.15468194	Q = 6.295249 P = 0.09809672	0.1253222	0.3619954
severity	GrimAge	Q = 2.164249 P = 0.3388748	Q = 2.572849 P = 0.4622694	0.03953384	0.6014878
severity	Hannum	Q = 5.522722 P = 6.769419	Q = 6.769419 P = 0.07962183	0.06785559	0.5708542
severity	HorvathAge	Q = 5.999045 P = 0.04981084	Q = 8.551572 P = 0.03588715	−0.1001956	0.453661
susceptibility	PhenoAge	Q = 11.42739 P = 0.02215828	Q = 14.92942 P = 0.01066820	−0.126466	0.3303144
susceptibility	GrimAge	Q = 4.090812 P = 0.3938553	Q = 4.147770 P = 0.5283430	−0.01238793	0.825028
susceptibility	Hannum	Q = 3.541134 P = 0.4716515	Q = 4.114188 P = 0.5330956	−0.03829989	0.4911899
susceptibility	HorvathAge	Q = 1.118961 P = 0.8912523	Q = 1.798353 P = 0.8762834	−0.04313577	0.456077
PhenoAge	severity	Q = 5.498383 P = 0.06397958	Q = 5.540632 P = 0.13622847	0.0130317	0.9126766
PhenoAge	susceptibility	Q = 6.542816 P = 0.2569210	Q = 6.951553 P = 0.3253508	−0.01010271	0.6003429
GrimAge	severity	NA	Q = 0.1205111 P = 0.7284808	NA	NA
GrimAge	susceptibility	Q = 0.1546549 P = 0.9255867	Q = 15.8688032 P = 0.0012064	0.3218178	0.05814232
Hannum	severity	NA	NA	NA	NA
Hannum	susceptibility	Q = 0.1054338 P = 0.9911775	Q = 0.1103816 P = 0.9985319	−0.001418437	0.9483488
HorvathAge	severity	Q = 15.77730 P = 0.2613651	Q = 15.92194 P = 0.3181648	0.007999366	0.7354521
HorvathAge	susceptibility	Q = 18.18951 P = 0.4432353	Q = 18.18961 P = 0.5098077	−6.61E-05	0.9921911

### Age has no significant effect on infection with severe COVID-19; severe COVID-19 can slow GrimAge acceleration

**FIGURE 3:**
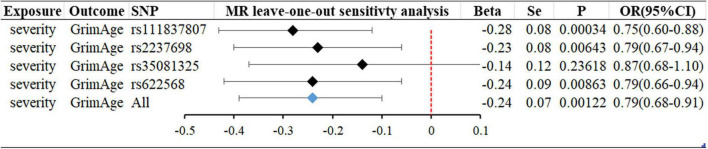
MR “leave-one-out” sensitivity analysis “COVID-19 severity” on “GrimAge.” “Leave-one-out” plot to visualize the causal effect of COVID-19 severity on the risk of GrimAge when leaving one SNP out.

In forwarding MR with epigenetic age as “exposure” and COVID-19 severity as “outcome,” the IVW meta-analysis showed that OR and 95%CI [PhenoAge and severity: 1.06 (0.91–1.23), *P* = 0.49; GrimAge and severity: 0.87 (0.72–1.05), *P* = 0.15; HorvathAge and severity: 0.97 (0.91–1.04), *P* = 0.36], the results were all of no significance. This suggests that epigenetic age doesn’t lead to increased severity of COVID-19 (Supplementary Figure 1). However, when performing the reverse causality assessment, COVID-19 severity had a negative causal relationship with GrimAge in the epigenetic age (severity and GrimAge: beta = −0.24, se = 0.07, *p* = 0.0012). The results remained statistically significant after the Bonferroni correction (*P* < 0.0125) (Supplementary Figure 3). This suggested that severe COVID-19 can slow GrimAge acceleration (Figure 3). The Cochran Q test for IVW (*P* = 0.46) and MR-Egger regression (*P* = 0.34) showed that there was no heterogeneity in SNPs. There was no significant statistical difference in egger_intercept and 0 of MR-Egger (*P* = 0.60), so we can assume that SNPs have no horizontal pleiotropy (Table 2). Mendelian randomization effect forest plot for a single SNP (Supplementary Figure 4).

### Telomeres do not affect susceptibility and severity of COVID-19

**FIGURE 4:**
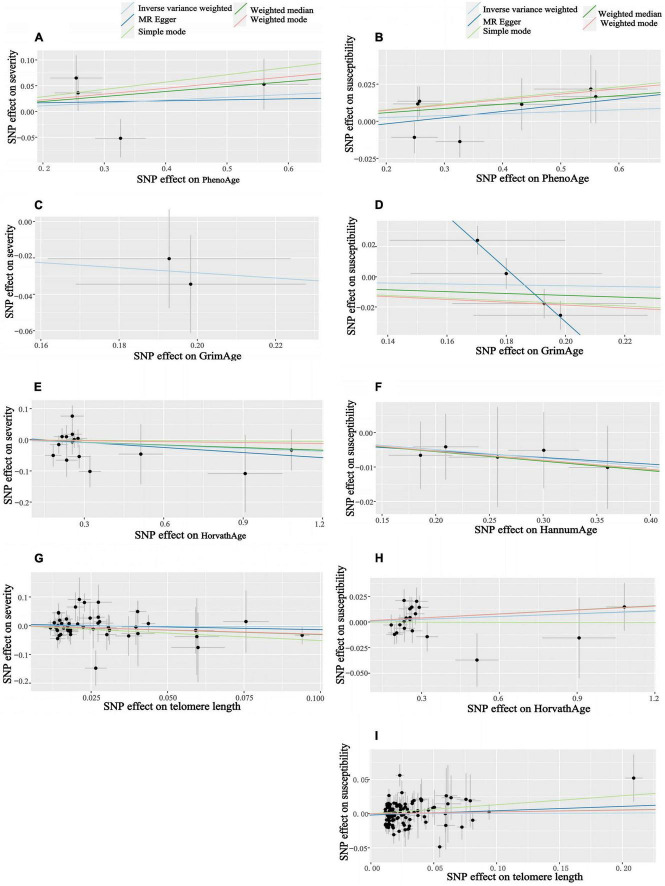
Scatter plots of aging and COVID-19 susceptibility and severity. Horizontal ordinate: SNP effect on “exposure”; Vertical coordinates: SNP effect on “outcome.” **(A)** Exposure: PhenoAge, outcome: severity; **(B)** exposure: PhenoAge, outcome: susceptibility; **(C)** exposure: GrimAge, outcome: severity; **(D)** exposure: GrimAge, outcome: susceptibility; **(E)** exposure: HorvathAge, outcome: severity; **(F)** exposure: HannumAge, outcome: susceptibility; **(G)** exposure: telomere length, outcome: severity; **(H)** exposure: HorvathAge, outcome: susceptibility; **(I)** exposure: telomere length, outcome: susceptibility.

When the telomere length is the exposure factor, susceptibility and severity of COVID-19 are “outcome.” The IVW results of the random effect model show no causal relationship between telomere length and COVID-19 susceptibility risk [OR (95%CI): 1.00 (0.91–1.11), *P* = 0.93) and severity risk (OR (95%CI): 0.97 (0.66–1.42), *P* = 0.86] (Figures 2, 4) (Supplementary Figure 5). Mendelian randomization effect forest plot for a single SNP (Supplementary Figure 6). The results of the MR-Egger regression of telomere length and COVID-19 susceptibility and severity showed that this result is unlikely to be affected by genetic pleiotropy (susceptibility: egger intercept = −0.0019, se = 0.0024, *P* = 0.42; severity: egger intercept = 0.0050, se = 0.0093, *P* = 0.59). Further, we observed no obvious heterogeneity between telomere length and COVID-19 severity (*Q* = 36.23, *P* = 0.87). Yet, there was heterogeneity between telomere length and COVID-19 susceptibility (*Q* = 125.76, *P* = 0.048), which might result from different genetic mechanisms (Table 3).

**TABLE 3:** Heterogeneity and pleiotropic tests for telomere length and susceptibility and severity of COVID-19.

		Q-statistics		Pleiotropic test	
EXPOSURE	OUTCOME	MR Egger	IVW	egger_intercept	pval
telomere length	severity	Q = 35.93498 P = 0.8569031	Q = 36.22570 P = 0.8727553	0.00499526	0.59236
telomere length	susceptibility	Q = 124.9386 P = 0.04635224	Q = 125.7620 P = 0.04812252	−0.001946896	0.4188337
severity	telomere length	Q = 0.3146255 P = 0.5748556	Q = 0.3270654 P = 0.8491387	0.000588234	0.9292872
susceptibility	telomere length	Q = 5.507894 P = 0.2390365	Q = 5.736572 P = 0.3327013	0.00151677	0.7044932

### Infection with severe COVID-19 can shorten telomere lengths

**FIGURE 5:**
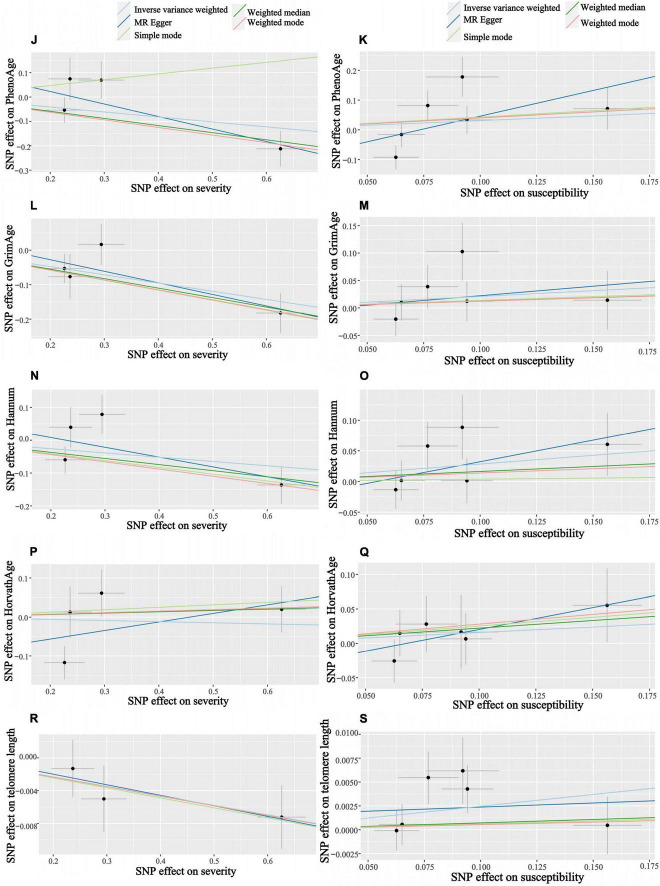
Scatter plots of COVID-19 susceptibility and severity and aging. Horizontal ordinate: SNP effect on “exposure”; Vertical coordinates: SNP effect on “outcome.” **(J)** Exposure: severity, outcome: PhenoAge; **(K)** exposure: susceptibility, outcome: PhenoAge; **(L)** exposure: severity, outcome: GrimAge; **(M)** exposure: susceptibility, outcome: GrimAge; **(N)** exposure: severity, outcome: HannumAge; **(O)** exposure: susceptibility, outcome: HannumAge; **(P)** exposure: severity, outcome: HorvathAge; **(Q)** exposure: susceptibility, outcome: HorvathAge; **(R)** exposure: severity, outcome: telomere length; **(S)** exposure: susceptibility, outcome: telomere length.

In reverse MR, the IVW results of the random-effect model showed a negative causal relationship between COVID-19 severity and telomere length [beta and 95%CI: −0.01 (−0.02, −0.001); se = 0.01; *P* = 0.02779], that is, infection with severe COVID-19 accelerates telomere wear and shortens telomere length (Figure 5). The Weighted median also showed similar results [beta and 95%CI: −0.01 (−0.02, −0.0006); se = 0.01; *P* = 0.03857] (Supplementary Figure 7). Then we performed heterogeneity analysis and intercept term analysis of MR-Egger regression, with no significant heterogeneity (*Q* = 0.33, P = 0.85) and pleiotropy (egger intercept = 0.00059, se = 0.0053, *P* = 0.93) (Table 3). Finally, we performed the “leave-one-out” analysis of the data using the IVW method, we removed one SNP in turn and analyzed the remaining SNP. We found that there was no SNP with a great impact on the results, and the results have important credibility (Supplementary Figure 8).

## Discussion

The link between aging, aging, and COVID-19 disease is a very novel area of research that has not yet been extensively studied. To some extent, epigenetic age and telomere length can be used as metrics of aging. When we looked at epigenetic age and telomere length versus susceptibility to COVID-19, there was insufficient evidence that aging was a predisposing factor for COVID-19, and there is no direct causal relationship between age stratification and COVID-19 infection. If aging has a causal effect on susceptibility to COVID-19, then its effect may be too small to be detected with our current sample size and a significance threshold of *P* = 0.05.

In our study, there is no forward causal relationship between aging and severe COVID-19 infection, i.e., the higher the risk of severe COVID-19 infection at age is debatable. But, the severity of COVID-19 has a negative causal relationship with the GrimAge clock of epigenetic age. That is, people with very severe respiratory symptoms of COVID-19 infection can delay the acceleration of epigenetic age (GrimAge) to some extent. Although only Grimage is significant out of the 4 clocks in epigenetic age and none of the rest is significant, however, this does not mean that our results are invalid. They may simply reflect differences in the way clocks were trained (e.g., training for different outcomes, tissues, and populations). Different clocks can provide insight into different biological mechanisms of aging (15). For example, GrimAge was trained on mortality and smoking (factors that are closely related to the risk of respiratory diseases), which may explain why it is superior to other epigenetic indicators of aging in studying respiratory diseases.

We analyze the reasons that severe COVID-19 may delay the epigenetic age GrimAge: (1) A longitudinal DNA methylation analysis showed that (21) while COVID-19 can have an impact on the epigenetic clock and telomere wear and tear and accelerate epigenetic aging, epigenetic age reversal occurs in some patients late in COVID-19 infection. In our “leave-one-out” analysis, we found that rs35081325 had a much greater impact on the results than other SNPs. Rs35081325 is located in the chr3p21 LZTFL1 intron sub-region, and studies have shown that (34–36) the chr3p21 region where rs35081325 is located is closely related to COVID severity. The secondary allele (allele “T”) of this gene has a protective effect. Epigenetic age reversal may occur because the population with the allele “A” decreases faster than the population with the protective allele “T” as the severity increases, resulting in a lower frequency of having the allele “A” in the surviving population. However, the data we used did not stratify the risk of severity in patients with COVID-19, and unfortunately, there are no biomarkers associated with aging that can be identified today as a risk stratification of severity in patients with COVID-19. (2) This may reflect that the association between aging and COVID-19 may be confounded by factors that are difficult to control for even with advanced statistical adjustments, like socioeconomic status, institutionalization, or various sub-health conditions of the body, or caused by insufficient statistical potency. (3) Epigenetic age and telomere length are not necessarily fully representative of aging. Studies have shown that p21CIP1, Ki-67, SA β-gal staining, p16INK4a, etc., can be used as biomarkers of aging. We must combine various biomarkers [e.g., morphological features, SA β-gal staining, p21CIP1, p16INK4a, heterochromatin markers (SAHFs and SADSs) and proliferation (Ki-67)] to accurately identify and confirm aging in cells (37). Using epigenetic age and telomere length alone to indicate aging is more limited.

When analyzing telomere length and COVID-19 severity, our results are similar to most observational studies (7–10), i.e., having severe COVID-19 infection can lead to accelerated telomere wear and shorter telomere length. Raul Sanchez-Vazquez et al. measured telomere length in 89 patients with COVID-19 and found that telomere length decreased with age, with patients with more severe COVID-19 having shorter telomere lengths compared to patients with milder COVID-19 (38). As we age, the accumulation of DNA damage affects the genome and chromosomal regions, with telomeres particularly vulnerable to age-related factors (39), and most mammalian somatic cells do not express telomerase, most types of somatic cells have limited ability to proliferate, a phenomenon known as replication senescence or “The Hayflick limit” (40, 41), which means that the telomere protective sequence that causes the ends of chromosomes is gradually lost as the number of replicates increases. The SARS-CoV-2 virus infects different cell types in the organism, and individuals with short telomeres suffer from impaired regeneration after infection with SARS-CoV-2, and these studies indirectly explain our findings (42).

## Conclusion

Our findings suggest that there is may no clear association between aging and susceptibility to COVID-19, and that COVID-19 severity is may not be associated with changes in age. Severe COVID-19 could lead to accelerated telomere wear and shorter telomere lengths. At the same time, severe COVID-19 could also slow the acceleration of GrimAge clocks, which is not significantly related to other epigenetic clocks. The fly in the ointment is that our study lacks observational studies, and existing observational studies differ from some of our findings. We conclude the above possible arguments through the Mendelian randomization approach. More research is needed to demonstrate the link between aging and adverse COVID-19 and the underlying mechanism by which this genetic predictive effect occurs.

## Author contributions

WX and FZ designed the study, performed data acquisition, analysis, interpretation, and drafted the manuscript. YS and YC performed data acquisition and analysis. BS and GY performed data interpretation. All authors read and approved the final manuscript.

## Footnotes

1
https://datashare.ed.ac.uk/handle/10283/3645
