# Supplementary material for: Causal association of epigenetic aging and COVID-19 severity and susceptibility: A bidirectional Mendelian randomization study
Source: Front Med (Lausanne). 2022 Sep 23;9:989950. doi: 10.3389/fmed.2022.989950 (PMC9538153; doi:10.3389/fmed.2022.989950)
Supplement: Supplementary file 1 [file Data_Sheet_1.pdf]

Causal association of aging and COVID-19 severity and susceptibility a bidirectional Mendelian randomization study

Supplementary Material.

This supplement contains 3 supplemental tables and 8 supplemental figure.

Supplementary Table 1. Epigenetic age instrumental variables and R^2 and F statistics

Exposure	SNP	EA	OA	EAF	Beta	Se	P value	R^2	R^2 combined	F
PhenoAge	rs1142345	T	C	0.95	0.82	0.09	3.35E-21	0.0002659	0.00437	152.18959
PhenoAge	rs7228835	C	G	0.12	-0.51	0.06	3.30E-17	0.000438		
PhenoAge	rs678553	T	C	0.69	0.33	0.04	2.27E-15	0.0007751		
PhenoAge	rs3829957	T	C	0.20	-0.38	0.05	3.51E-15	0.0005662		
PhenoAge	rs752223	A	G	0.08	-0.56	0.07	7.20E-15	0.000262		
PhenoAge	rs1990053	A	G	0.42	0.26	0.04	2.05E-11	0.0006314		
PhenoAge	rs11190127	A	C	0.38	0.25	0.04	3.83E-10	0.0005307		
PhenoAge	rs6531114	T	C	0.26	-0.25	0.04	2.07E-09	0.0004017		
PhenoAge	rs11253338	T	C	0.18	0.28	0.05	8.49E-09	0.0002871		
PhenoAge	rs116853700	A	G	0.04	0.55	0.10	1.59E-08	7.492E-05		
PhenoAge	rs73028070	A	G	0.08	-0.43	0.08	1.74E-08	0.0001326		
GrimAge	rs9386796	T	C	0.46	0.20	0.03	1.64E-11	0.0006511	0.00202	70.34253
GrimAge	rs887466	A	G	0.38	-0.19	0.03	5.09E-10	0.0005259		
GrimAge	rs4065321	T	C	0.53	-0.17	0.03	8.78E-09	0.0004747		
GrimAge	rs17094148	A	G	0.71	-0.18	0.03	2.55E-08	0.0003709		
Hannum	rs10786282	A	G	0.21	-0.36	0.04	2.61E-23	0.0009533	0.00446	155.42771
Hannum	rs1005277	A	C	0.30	0.30	0.03	8.92E-20	0.0010006		
Hannum	rs12417758	T	C	0.55	-0.21	0.03	6.22E-12	0.000677		
Hannum	rs1598856	A	G	0.45	0.19	0.03	3.63E-10	0.0005617		
Hannum	rs3093956	T	C	0.81	-0.24	0.04	8.39E-10	0.0003277		
Hannum	rs4383328	A	T	0.28	-0.19	0.03	9.57E-09	0.0003869		
Hannum	rs111731678	A	T	0.19	-0.23	0.04	1.26E-08	0.0002919		
Hannum	rs4838595	T	C	0.12	-0.26	0.05	1.29E-08	0.0001992		
Hannum	rs34970912	C	G	0.96	-0.52	0.09	2.88E-08	6.031E-05		
HorvathAge	rs10949481	A	T	0.95	1.08	0.07	4.56E-54	0.0006958	0.01259	442.37803
HorvathAge	rs3917672	A	G	0.49	-0.26	0.03	5.95E-18	0.0010707	0.01260	
HorvathAge	rs57941717	T	G	0.25	0.29	0.04	3.71E-16	0.0007222		
HorvathAge	rs10447389	A	G	0.27	-0.28	0.03	4.42E-16	0.0007483		
HorvathAge	rs6414374	A	G	0.16	0.32	0.04	1.42E-14	0.0004636		
HorvathAge	rs4240228	T	G	0.72	0.25	0.03	2.47E-14	0.0006802		
HorvathAge	rs10732882	T	G	0.41	-0.24	0.03	3.68E-14	0.0007947		
HorvathAge	rs7550821	T	C	0.24	-0.26	0.04	3.27E-13	0.0005648		
HorvathAge	rs7627756	A	G	0.56	0.22	0.03	6.28E-13	0.0007319		
HorvathAge	rs1511762	T	C	0.22	0.26	0.04	1.53E-12	0.0004912		
HorvathAge	rs2736099	A	G	0.35	0.23	0.03	3.47E-12	0.0006389		

HorvathAge	rs12043492	T	C	0.42	0.22	0.03	4.35E-12	0.0006727
HorvathAge	rs75243280	T	C	0.67	-0.23	0.03	7.37E-12	0.0005995
HorvathAge	rs2492286	T	G	0.15	0.28	0.04	5.29E-11	0.0003195
HorvathAge	rs6577536	A	G	0.48	0.20	0.03	6.73E-11	0.0006111
HorvathAge	rs79111787	T	C	0.97	-0.91	0.14	1.28E-10	7.419E-05
HorvathAge	rs12903325	T	G	0.76	-0.22	0.04	5.48E-10	0.0004054
HorvathAge	rs1726672	T	C	0.30	-0.20	0.03	6.05E-10	0.0004675
HorvathAge	rs144317085	A	T	0.96	0.51	0.08	6.42E-10	8.209E-05
HorvathAge	rs10735418	T	C	0.63	0.20	0.03	1.14E-09	0.0004989
HorvathAge	rs1488106	T	C	0.37	0.18	0.03	4.25E-09	0.0004646
HorvathAge	rs12666349	T	C	0.80	0.25	0.04	5.23E-09	0.0003115
HorvathAge	rs2275558	A	G	0.22	-0.23	0.04	6.44E-09	0.0003349
HorvathAge	rs34003787	T	C	0.09	0.32	0.06	2.54E-08	0.0001411

*SNP: Single Nucleotide Polymorphism; EA: Effect Allele; OA: Other Allele; EAF: Effector Allele Frequency; Beta: SNP effect size on exposure; Se: standard error; P: P-value; R^2 indicates the extent to which instrumental variables explain exposure (coefficient of determination of the regression equation); R^2 combined: Sum of R^2 of all SNPs; F: F-statistic

Supplementary Table 2. Telomere length instrumental variables and R^2 and F statistics

Exposure	SNP	EA	OA	EAF	Beta	Se	P value	R^2	R^2combined	F
telomere length	rs3767952	A	G	0.23	0.01	0.00	1.80E-08	2.35E-05	0.01181	36.63
telomere length	rs4498805	T	G	0.55	0.02	0.00	5.70E-14	5.93E-05		
telomere length	rs932002	T	C	0.15	-0.04	0.00	7.30E-47	1.12E-04		
telomere length	rs66731853	A	G	0.32	-0.02	0.00	1.50E-16	6.25E-05		
telomere length	rs11584821	T	C	0.18	-0.03	0.00	3.00E-31	8.31E-05		
telomere length	rs6659669	T	C	0.61	-0.01	0.00	1.10E-08	3.30E-05		
telomere length	rs2977608	C	A	0.74	0.01	0.00	3.00E-08	2.48E-05		
telomere length	rs6669563	A	G	0.44	0.02	0.00	2.10E-19	8.46E-05		
telomere length	rs145114957	G	C	0.04	0.03	0.00	4.60E-08	5.16E-06		
telomere length	rs41269079	A	T	0.19	0.02	0.00	1.70E-09	2.36E-05		
telomere length	rs139795227	C	A	0.01	0.06	0.01	6.70E-12	2.76E-06		
telomere length	rs11579626	C	A	0.08	0.03	0.00	1.30E-13	1.81E-05		
telomere length	rs6587577	G	A	0.83	-0.02	0.00	4.80E-12	2.90E-05		
telomere length	rs6751209	C	T	0.20	-0.01	0.00	1.60E-08	2.20E-05		
telomere length	rs188918174	T	C	0.04	0.04	0.01	1.20E-13	8.10E-06		
telomere length	rs202034370	T	TA	0.98	0.10	0.01	2.60E-56	2.54E-05		
telomere length	rs56178008	A	T	0.44	0.01	0.00	9.70E-13	5.31E-05		
telomere length	rs376641875	C	CATAA	0.93	-0.03	0.00	3.30E-10	1.11E-05		
telomere length	rs17803849	T	C	0.41	0.03	0.00	4.20E-41	1.84E-04		
telomere length	rs2555104	C	A	0.43	-0.01	0.00	6.60E-12	4.91E-05		
telomere length	rs77732866	A	G	0.14	0.02	0.00	9.20E-10	1.88E-05		
telomere length	rs2230590	C	T	0.51	-0.02	0.00	3.60E-15	6.55E-05		
telomere length	rs78491606	C	A	0.02	-0.08	0.01	1.90E-24	7.98E-06		
telomere length	rs9878436	T	C	0.43	-0.01	0.00	1.20E-12	5.25E-05		
telomere length	rs35446936	A	G	0.24	-0.09	0.00	1.00E-200	1.27E-03		
telomere length	rs112394943	C	T	0.16	-0.02	0.00	1.60E-12	2.88E-05		
telomere length	rs13062095	C	T	0.33	0.01	0.00	9.70E-11	3.91E-05		
telomere length	rs4616688	T	G	0.53	-0.02	0.00	4.50E-18	7.93E-05		
telomere length	rs6790988	G	A	0.74	0.01	0.00	1.80E-10	3.30E-05		
telomere length	rs869785	C	T	0.67	-0.01	0.00	4.40E-12	4.47E-05		
telomere length	rs6776756	A	G	0.60	-0.02	0.00	1.10E-17	7.47E-05		
telomere length	rs11426156	T	TA	0.40	-0.01	0.00	2.20E-08	3.18E-05		
telomere length	rs35500378	CACTT	C	0.61	0.01	0.00	2.00E-12	4.99E-05		
telomere length	rs10805346	C	T	0.44	0.01	0.00	7.00E-09	3.50E-05		
telomere length	rs6536702	A	G	0.77	0.05	0.00	9.40E-111	3.70E-04		
telomere length	rs871134	T	C	0.57	-0.02	0.00	1.70E-19	8.47E-05		
telomere length	rs4695407	G	A	0.51	0.01	0.00	1.50E-12	5.30E-05		
telomere length	rs2282764	G	A	0.14	-0.02	0.00	9.30E-15	3.11E-05		
telomere length	rs7705526	A	C	0.33	0.08	0.00	1.00E-200	1.20E-03		
telomere length	rs73730598	A	G	0.05	0.03	0.00	4.70E-10	8.51E-06		

telomere length	rs61748181	T	C	0.03	-0.06	0.01	2.80E-23	1.18E-05
telomere length	rs6881568	A	C	0.36	0.02	0.00	3.70E-16	6.50E-05
telomere length	rs55747751	A	G	0.08	-0.02	0.00	1.70E-08	9.62E-06
telomere length	rs185174247	A	G	0.06	0.04	0.00	1.10E-17	1.65E-05
telomere length	rs141214782	TTATC	T	0.10	-0.02	0.00	2.00E-13	2.08E-05
telomere length	rs28363070	A	G	0.01	0.08	0.01	3.50E-15	3.47E-06
telomere length	rs1611236	A	G	0.33	-0.02	0.00	6.10E-14	5.25E-05
telomere length	rs142730696	TTTTTC	T	0.86	0.02	0.00	6.30E-13	2.57E-05
telomere length	rs7772289	T	G	0.50	0.02	0.00	1.70E-18	8.15E-05
telomere length	rs2763979	T	C	0.36	-0.03	0.00	1.30E-40	1.74E-04
telomere length	rs80324517	A	G	0.05	0.04	0.00	1.80E-17	1.41E-05
telomere length	rs201558190	C	T	0.36	-0.02	0.00	6.40E-17	6.84E-05
telomere length	rs9398196	G	A	0.52	-0.01	0.00	9.50E-13	5.39E-05
telomere length	rs61405042	T	C	0.03	-0.05	0.01	8.50E-17	8.34E-06
telomere length	rs2538745	C	T	0.60	-0.01	0.00	3.10E-10	4.02E-05
telomere length	rs117407747	T	C	0.03	0.05	0.01	1.80E-13	6.16E-06
telomere length	rs1985369	G	A	0.87	-0.03	0.00	3.60E-25	5.21E-05
telomere length	rs13230646	C	T	0.25	-0.02	0.00	8.90E-14	4.40E-05
telomere length	rs11769630	A	T	0.07	-0.03	0.00	4.30E-11	1.23E-05
telomere length	rs2056726	A	G	0.21	-0.02	0.00	7.90E-21	6.25E-05
telomere length	rs7790856	T	C	0.29	-0.04	0.00	1.80E-87	3.42E-04
telomere length	rs4731541	G	C	0.62	-0.02	0.00	1.40E-23	9.94E-05
telomere length	rs117630647	A	G	0.02	0.06	0.01	1.40E-16	6.05E-06
telomere length	rs2306646	C	G	0.56	-0.02	0.00	3.30E-25	1.12E-04
telomere length	rs762679	A	T	0.86	0.03	0.00	1.40E-27	6.16E-05
telomere length	rs11991877	A	T	0.89	-0.03	0.00	3.20E-21	3.73E-05
telomere length	rs10112752	A	G	0.43	-0.03	0.00	9.50E-46	2.09E-04
telomere length	rs1023767	A	G	0.24	-0.02	0.00	5.00E-15	4.70E-05
telomere length	rs34896435	G	C	0.47	0.02	0.00	6.40E-14	5.93E-05
telomere length	rs11557154	T	C	0.13	-0.03	0.00	1.10E-30	6.35E-05
telomere length	rs4743037	T	C	0.23	0.01	0.00	5.10E-10	2.91E-05
telomere length	rs117034449	A	G	0.02	0.04	0.01	2.10E-08	3.03E-06
telomere length	rs7099229	A	G	0.27	-0.02	0.00	8.40E-12	3.93E-05
telomere length	rs12412214	A	G	0.28	-0.02	0.00	3.40E-28	1.03E-04
telomere length	rs6584579	G	A	0.40	0.01	0.00	2.00E-08	3.20E-05
telomere length	rs9419958	C	T	0.86	-0.08	0.00	2.60E-167	3.84E-04
telomere length	rs77231040	C	G	0.01	0.10	0.01	2.00E-13	1.31E-06
telomere length	rs10905255	T	G	0.58	-0.02	0.00	2.60E-19	8.34E-05
telomere length	rs939916	A	G	0.67	0.02	0.00	6.60E-29	1.17E-04
telomere length	rs10840270	G	C	0.66	0.01	0.00	1.30E-11	4.38E-05
telomere length	rs2293579	A	G	0.39	-0.01	0.00	3.30E-10	3.97E-05
telomere length	rs10768683	G	C	0.84	0.05	0.00	1.50E-64	1.63E-04

telomere length	rs11212631	C	T	0.20	-0.02	0.00	4.70E-14	3.84E-05
telomere length	rs6590343	G	A	0.52	0.01	0.00	1.50E-09	3.86E-05
telomere length	rs611646	A	T	0.41	-0.04	0.00	3.50E-73	3.35E-04
telomere length	rs12369950	C	T	0.14	-0.02	0.00	8.00E-10	1.93E-05
telomere length	rs79977579	A	C	0.10	0.03	0.00	2.30E-16	2.46E-05
telomere length	rs10773176	G	A	0.74	-0.02	0.00	5.20E-14	4.60E-05
telomere length	rs10845387	A	G	0.35	-0.01	0.00	1.50E-11	4.40E-05
telomere length	rs17445108	A	G	0.13	-0.02	0.00	2.00E-08	1.48E-05
telomere length	rs10774624	A	G	0.53	0.01	0.00	2.90E-13	5.61E-05
telomere length	rs1907702	A	G	0.77	0.02	0.00	5.90E-10	2.90E-05
telomere length	rs76666449	C	T	0.10	0.03	0.00	8.20E-19	3.01E-05
telomere length	rs28577594	C	G	0.71	0.02	0.00	5.40E-17	6.12E-05
telomere length	rs1332941	G	A	0.82	0.03	0.00	5.90E-21	5.50E-05
telomere length	rs670180	A	T	0.57	-0.01	0.00	1.20E-08	3.38E-05
telomere length	rs9600019	T	C	0.34	0.01	0.00	2.40E-09	3.36E-05
telomere length	rs73581419	T	C	0.11	0.02	0.00	1.30E-12	2.03E-05
telomere length	rs113525195	A	C	0.29	-0.01	0.00	3.10E-08	2.67E-05
telomere length	rs45604339	T	C	0.34	-0.02	0.00	4.30E-22	8.91E-05
telomere length	rs137901416	A	G	0.10	0.05	0.00	4.70E-43	7.23E-05
telomere length	rs1957937	T	A	0.16	0.02	0.00	1.90E-14	3.34E-05
telomere length	rs3093888	A	G	0.05	-0.03	0.00	1.50E-10	8.45E-06
telomere length	rs34550383	C	CT	0.55	-0.02	0.00	8.60E-22	9.66E-05
telomere length	rs17677991	G	C	0.34	0.02	0.00	4.40E-26	1.06E-04
telomere length	rs5742915	C	T	0.45	0.02	0.00	1.60E-21	9.51E-05
telomere length	rs7164950	G	A	0.41	0.01	0.00	2.30E-10	4.11E-05
telomere length	rs11412296	T	TA	0.76	0.03	0.00	1.40E-45	1.55E-04
telomere length	rs80116508	A	G	0.06	-0.04	0.00	2.00E-17	1.79E-05
telomere length	rs76219171	A	G	0.06	0.04	0.00	7.80E-17	1.62E-05
telomere length	rs2967355	C	A	0.77	-0.05	0.00	4.00E-83	2.76E-04
telomere length	rs12925933	C	A	0.66	-0.01	0.00	7.00E-12	4.46E-05
telomere length	rs111950327	C	G	0.06	0.02	0.00	5.90E-09	8.55E-06
telomere length	rs450962	G	A	0.28	0.01	0.00	5.90E-09	2.92E-05
telomere length	rs3785074	G	A	0.29	0.02	0.00	2.60E-27	1.02E-04
telomere length	rs76065543	T	C	0.14	0.03	0.00	4.20E-32	6.99E-05
telomere length	rs12932179	G	A	0.56	-0.01	0.00	1.80E-11	4.71E-05
telomere length	rs182059586	C	T	0.03	-0.06	0.01	4.90E-17	7.30E-06
telomere length	rs11117354	C	T	0.70	0.02	0.00	3.40E-26	1.00E-04
telomere length	rs9940099	T	G	0.06	-0.03	0.00	3.20E-16	1.66E-05
telomere length	rs56061761	A	G	0.33	-0.02	0.00	6.90E-20	7.84E-05
telomere length	rs4724	A	G	0.12	-0.05	0.00	9.80E-69	1.34E-04
telomere length	rs12451892	C	T	0.38	-0.01	0.00	2.20E-08	3.13E-05
telomere length	rs7209057	A	G	0.56	0.01	0.00	5.70E-09	3.54E-05
telomere length	rs59409453	G	A	0.73	0.02	0.00	1.60E-18	6.43E-05
telomere length	rs111527438	C	T	0.35	0.01	0.00	3.10E-09	3.39E-05

telomere length	rs7221585	T	C	0.22	0.01	0.00	6.70E-09	2.48E-05
telomere length	rs75664430	G	C	0.25	-0.02	0.00	3.60E-24	8.13E-05
telomere length	rs56799554	G	A	0.17	-0.03	0.00	3.00E-22	5.63E-05
telomere length	rs144204502	T	C	0.01	-0.10	0.01	3.40E-28	6.37E-06
telomere length	rs150150565	T	C	0.02	0.06	0.01	6.80E-18	6.60E-06
telomere length	rs116863223	A	G	0.01	-0.08	0.01	2.60E-18	3.75E-06
telomere length	rs16978028	T	A	0.14	-0.03	0.00	8.20E-26	5.75E-05
telomere length	rs2276182	G	C	0.40	0.02	0.00	2.80E-30	1.33E-04
telomere length	rs9955360	A	C	0.87	-0.02	0.00	2.20E-10	1.94E-05
telomere length	rs3891167	G	A	0.25	-0.04	0.00	1.20E-70	2.53E-04
telomere length	rs139669835	T	C	0.01	-0.06	0.01	6.10E-09	1.33E-06
telomere length	rs11085072	T	C	0.24	-0.01	0.00	2.60E-08	2.37E-05
telomere length	rs4530278	T	G	0.60	0.01	0.00	1.50E-11	4.64E-05
telomere length	rs8105767	G	A	0.29	0.03	0.00	2.50E-50	1.96E-04
telomere length	rs429358	C	T	0.15	0.02	0.00	3.80E-10	2.16E-05
telomere length	rs8102497	A	G	0.43	-0.01	0.00	1.40E-13	5.69E-05
telomere length	rs11699829	A	G	0.03	0.06	0.01	1.50E-26	1.59E-05
telomere length	rs142426306	T	C	0.04	-0.05	0.01	8.70E-21	1.41E-05
telomere length	rs1291143	C	A	0.85	0.05	0.00	1.80E-69	1.69E-04
telomere length	rs6054257	A	G	0.79	-0.01	0.00	1.10E-08	2.27E-05
telomere length	rs35640778	A	G	0.02	-0.21	0.01	9.59E-195	7.63E-05
telomere length	rs143190905	T	G	0.08	-0.07	0.00	1.60E-85	1.20E-04
telomere length	rs41304832	A	G	0.01	0.06	0.01	5.00E-11	2.24E-06
telomere length	rs117512405	A	G	0.02	-0.08	0.01	9.50E-22	6.51E-06
telomere length	rs28502153	A	C	0.38	-0.02	0.00	1.20E-25	1.09E-04
telomere length	rs6007020	C	T	0.37	0.01	0.00	4.80E-12	4.71E-05
telomere length	rs131797	T	TAAAAA	0.24	0.02	0.00	6.80E-25	8.10E-05
telomere length	rs1003322	A	C	0.21	0.01	0.00	1.00E-08	2.33E-05

*SNP: Single Nucleotide Polymorphism; EA: Effect Allele; OA: Other Allele; EAF: Effector Allele Frequency; Beta: SNP effect size on exposure; Se: standard error; P: P-value; R^2 indicates the extent to which instrumental variables explain exposure (coefficient of determination of the regression equation); R^2 combined: Sum of R^2 of all SNPs; F: F-statistic

Supplementary Table 3. COVID-19 susceptibility and severity instrumental variables and R^2, F statistics

**Exposure**	**SNP**	**EA**	**OA**	**MAF**	**Beta**	**Se**	**P value**	**R^2**	**R^2combined**	**F**
severity	rs35081325	A	T	0.08	0.63	0.04	5.75E-45	1.99E-05	3.69E-05	51.20
severity	rs111837807	T	C	0.10	0.29	0.04	5.66E-12	6.13E-06		
severity	rs622568	A	C	0.15	0.23	0.04	1.04E-09	6.70E-06		
severity	rs2237698	C	T	0.09	0.24	0.04	2.41E-09	4.19E-06		
susceptibility	rs4971066	T	G	0.18	-0.08	0.01	1.02E-08	5.70E-06	5.61E-05	94.54
susceptibility	rs2271616	G	T	0.12	0.16	0.02	3.61E-25	1.33E-05		
susceptibility	rs17078348	A	G	0.10	0.09	0.02	1.20E-08	3.46E-06		
susceptibility	rs10936744	C	T	0.36	-0.06	0.01	3.51E-10	1.08E-05		
susceptibility	rs579459	C	T	0.79	-0.09	0.01	2.18E-16	1.33E-05		
susceptibility	rs10744789	T	C	0.72	0.07	0.01	2.72E-10	9.65E-06		

*SNP: Single Nucleotide Polymorphism; EA: Effect Allele; OA: Other Allele; MAF: Minor Allele Frequency; Beta: SNP effect size on exposure; Se: standard error; P: P-value; R^2 indicates the extent to which instrumental variables explain exposure (coefficient of determination of the regression equation); R^2 combined: Sum of R^2 of all SNPs; F: F-statistic

Supplementary Figure 1. OR estimates and 95% CI for five methods with epigenetic age as the exposure and COVID-19 susceptibility and severity as the outcome.

Exposure	Outcome	Method	nSNP	Beta	Se	P	OR(95%CI)
phenoAge	severity	MR Egger	4	0.02	0.30	0.95584	1.02(0.56-1.85)
phenoAge	severity	Weighted median	4	0.10	0.08	0.20684	1.10(0.95-1.28)
phenoAge	severity	IVW	4	0.05	0.08	0.48502	1.06(0.91-1.23)
phenoAge	severity	Simple mode	4	0.14	0.11	0.28377	1.15(0.93-1.43)
phenoAge	severity	Weighted mode	4	0.11	0.09	0.30802	1.12(0.93-1.34)
phenoAge	susceptibility	MR Egger	7	0.04	0.05	0.47662	1.04(0.94-1.16)
phenoAge	susceptibility	Weighted median	7	0.03	0.02	0.13871	1.03(0.99-1.07)
phenoAge	susceptibility	IVW	7	0.01	0.02	0.40700	1.01(0.98-1.04)
phenoAge	susceptibility	Simple mode	7	0.04	0.03	0.29394	1.04(0.97-1.11)
phenoAge	susceptibility	Weighted mode	7	0.04	0.03	0.24837	1.04(0.98-1.10)
GrimAge	severity	IVW	2	-0.14	0.10	0.14699	0.87(0.72-1.05)
GrimAge	susceptibility	MR Egger	4	-1.76	0.44	0.05654	0.17(0.07-0.41)
GrimAge	susceptibility	Weighted median	4	-0.06	0.03	0.07542	0.94(0.88-1.01)
GrimAge	susceptibility	IVW	4	-0.03	0.06	0.62192	0.97(0.86-1.09)
GrimAge	susceptibility	Simple mode	4	-0.09	0.06	0.21598	0.92(0.82-1.02)
GrimAge	susceptibility	Weighted mode	4	-0.09	0.04	0.10631	0.91(0.84-0.99)

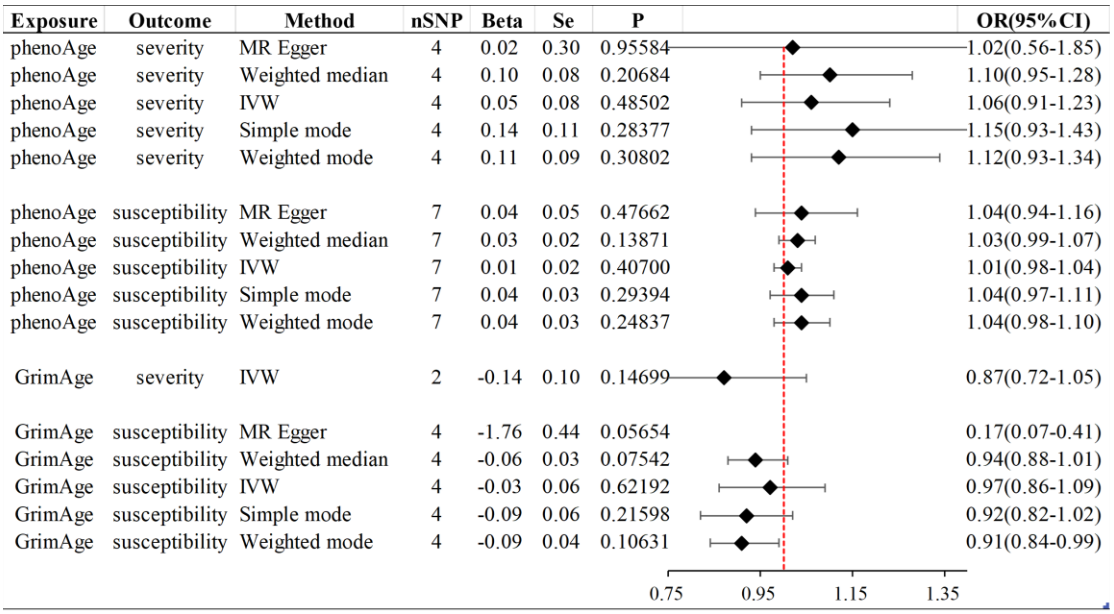

Exposure	Outcome	Method	nSNP	Beta	Se	P	OR(95%CI)
Hannum	severity	Wald ratio	1	-0.26	0.14	0.06817	0.77(0.58-1.02)
Hannum	susceptibility	MR Egger	5	-0.02	0.08	0.81894	0.98(0.84-1.14)
Hannum	susceptibility	Weighted median	5	-0.03	0.02	0.20077	0.97(0.93-1.01)
Hannum	susceptibility	IVW	5	-0.02	0.02	0.19246	0.98(0.94-1.01)
Hannum	susceptibility	Simple mode	5	-0.03	0.03	0.40868	0.97(0.92-1.03)
Hannum	susceptibility	Weighted mode	5	-0.03	0.03	0.37682	0.97(0.92-1.03)
HorvathAge	severity	MR Egger	15	-0.05	0.08	0.49249	0.95(0.81-1.10)
HorvathAge	severity	Weighted median	15	-0.03	0.05	0.54694	0.97(0.89-1.06)
HorvathAge	severity	IVW	15	-0.03	0.03	0.36188	0.97(0.91-1.04)
HorvathAge	severity	Simple mode	15	0.00	0.07	0.95212	1.00(0.87-1.14)
HorvathAge	severity	Weighted mode	15	-0.01	0.05	0.84305	0.99(0.90-1.09)
HorvathAge	susceptibility	MR Egger	20	0.01	0.02	0.70664	1.01(0.96-1.06)
HorvathAge	susceptibility	Weighted median	20	0.01	0.01	0.32241	1.01(0.99-1.04)
HorvathAge	susceptibility	IVW	20	0.01	0.01	0.34336	1.01(0.99-1.03)
HorvathAge	susceptibility	Simple mode	20	0.00	0.02	0.98516	1.00(0.95-1.05)
HorvathAge	susceptibility	Weighted mode	20	0.01	0.02	0.50259	1.01(0.98-1.05)

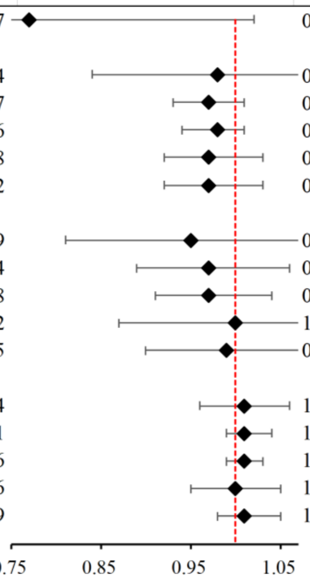

*Methods: MR Egger、Weighted median、IVW、Simple mode、Weighted mode; Beta: risk index; Se: standard error; P: P-value; OR(95%CI):odds ratio(95%confidence interval); Forest plot: Visualize the causal effect of epigenetic age on the risk of COVID-19 susceptibility and severity by five methods(The standard line is the line of "X=1" (red dashed line))

Supplementary Figure 2. Mendelian randomization effects forest plots for individual SNPs with epigenetic age as the exposure, COVID-19 susceptibility and severity as the outcome

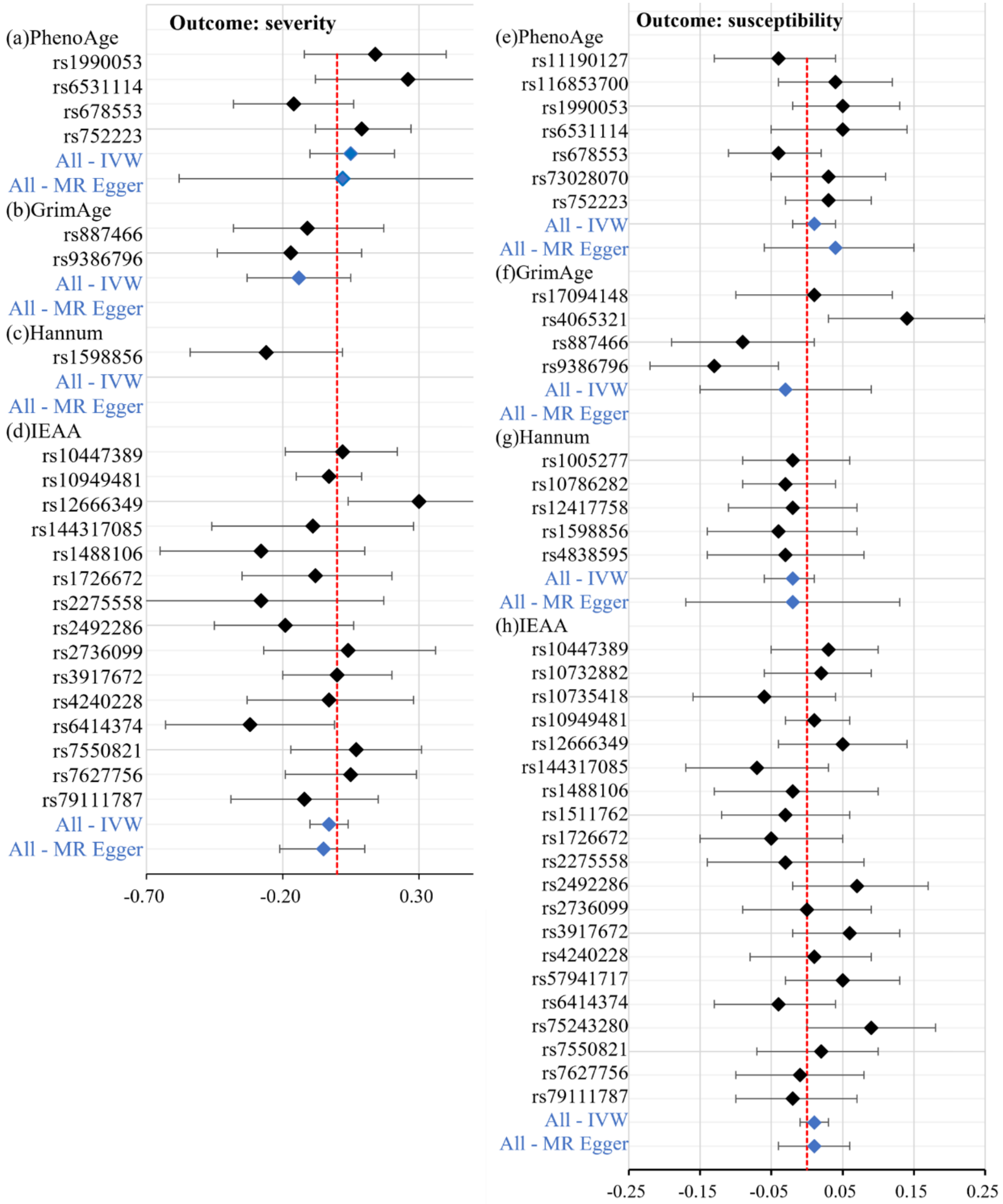

* This figure showed the forest plots for individual SNPs with epigenetic age as the exposure, COVID-19 susceptibility and severity as the outcome. (a)exposure: PhenoAge, outcome: susceptibility; (b)exposure: GrimAge, outcome: susceptibility; (c)exposure: Hannum, outcome: susceptibility; (d)exposure: HorvathAge, outcome: susceptibility; (e)exposure: PhenoAge, outcome: severity; (f)exposure: GrimAge, outcome: severity; (g)exposure: HannumAge, outcome: severity; (h)exposure: HorvathAge, outcome: severity; The blue marker points indicated All-IVW and All-MR Egger

Supplementary Figure 3. OR estimates and 95% CI of the five methods with COVID-19 susceptibility and severity as the exposure and epigenetic age as the outcome

Exposure	Outcome	Method	nSNP	Beta	Se	P	OR(95%CI)
severity	PhenoAge	MR Egger	4	-0.51	0.29	0.22211	0.60(0.34-1.06)
severity	PhenoAge	Weighted median	4	-0.29	0.11	0.00689	0.75(0.60-0.92)
severity	PhenoAge	IVW	4	-0.20	0.14	0.13183	0.82(0.63-1.06)
severity	PhenoAge	Simple mode	4	0.24	0.24	0.39303	1.27(0.79-2.02)
severity	PhenoAge	Weighted mode	4	-0.31	0.11	0.06764	0.73(0.59-0.91)
severity	GrimAge	MR Egger	4	-0.34	0.18	0.19731	0.71(0.51-1.01)
severity	GrimAge	Weighted median	4	-0.27	0.08	0.00089	0.76(0.65-0.89)
severity	GrimAge	IVW	4	-0.24	0.07	0.00122	0.79(0.68-0.91)
severity	GrimAge	Simple mode	4	-0.29	0.13	0.10855	0.75(0.58-0.96)
severity	GrimAge	Weighted mode	4	-0.29	0.09	0.05133	0.75(0.63-0.90)
severity	Hannum	MR Egger	4	-0.30	0.28	0.39615	0.74(0.43-1.28)
severity	Hannum	Weighted median	4	-0.19	0.08	0.02624	0.83(0.71-0.98)
severity	Hannum	IVW	4	-0.13	0.11	0.23469	0.88(0.71-1.09)
severity	Hannum	Simple mode	4	-0.21	0.17	0.31230	0.81(0.58-1.14)
severity	Hannum	Weighted mode	4	-0.22	0.08	0.07727	0.80(0.68-0.94)
severity	HorvathAge	MR Egger	4	0.22	0.30	0.53937	1.25(0.69-2.24)
severity	HorvathAge	Weighted median	4	0.03	0.09	0.69790	1.03(0.87-1.23)
severity	HorvathAge	IVW	4	-0.03	0.13	0.81810	0.97(0.76-1.24)
severity	HorvathAge	Simple mode	4	0.06	0.14	0.68962	1.07(0.80-1.41)
severity	HorvathAge	Weighted mode	4	0.04	0.09	0.69146	1.04(0.87-1.23)

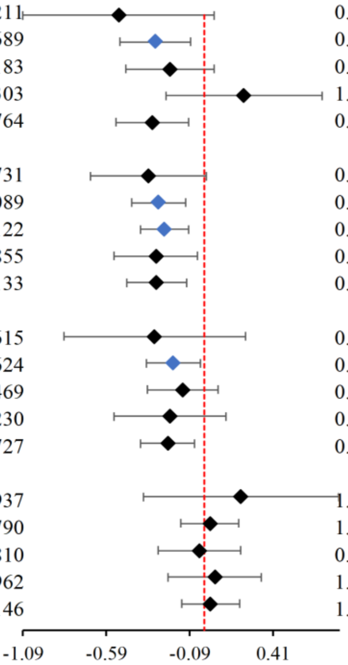

Exposure	Outcome	Method	nSNP	Beta	Se	P	OR(95%CI)
susceptibility	PhenoAge	MR Egger	6	1.73	1.34	0.26650	5.62(0.41-77.28)
susceptibility	PhenoAge	Weighted median	6	0.40	0.32	0.20481	1.49(0.80-2.78)
susceptibility	PhenoAge	IVW	6	0.31	0.41	0.44326	1.37(0.62-3.03)
susceptibility	PhenoAge	Simple mode	6	0.43	0.46	0.39688	1.54(0.62-3.82)
susceptibility	PhenoAge	Weighted mode	6	0.40	0.36	0.31766	1.50(0.73-3.04)
susceptibility	GrimAge	MR Egger	6	0.35	0.61	0.59993	1.41(0.43-4.66)
susceptibility	GrimAge	Weighted median	6	0.12	0.23	0.59146	1.13(0.72-1.78)
susceptibility	GrimAge	IVW	6	0.21	0.18	0.25203	1.23(0.86-1.76)
susceptibility	GrimAge	Simple mode	6	0.14	0.30	0.66165	1.15(0.64-2.05)
susceptibility	GrimAge	Weighted mode	6	0.12	0.26	0.66341	1.13(0.67-1.90)
susceptibility	Hannum	MR Egger	6	0.70	0.58	0.29401	2.02(0.64-6.36)
susceptibility	Hannum	Weighted median	6	0.16	0.22	0.46603	1.18(0.76-1.82)
susceptibility	Hannum	IVW	6	0.28	0.18	0.11407	1.33(0.93-1.89)
susceptibility	Hannum	Simple mode	6	0.04	0.33	0.91632	1.04(0.54-1.99)
susceptibility	Hannum	Weighted mode	6	0.14	0.30	0.66216	1.15(0.64-2.06)
susceptibility	HorvathAge	MR Egger	6	0.63	0.61	0.35443	1.88(0.57-6.18)
susceptibility	HorvathAge	Weighted median	6	0.22	0.23	0.33254	1.25(0.80-1.95)
susceptibility	HorvathAge	IVW	6	0.16	0.19	0.39275	1.17(0.81-1.69)
susceptibility	HorvathAge	Simple mode	6	0.25	0.31	0.45122	1.29(0.70-2.36)
susceptibility	HorvathAge	Weighted mode	6	0.28	0.28	0.36568	1.32(0.76-2.30)

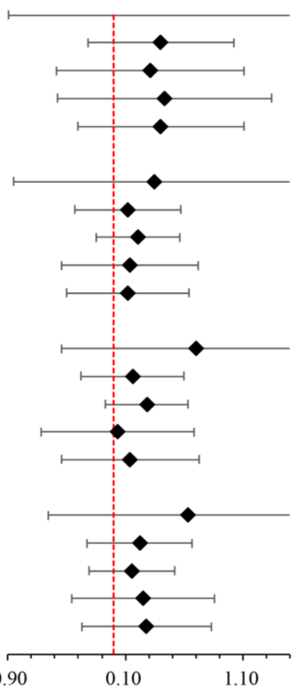

*Methods: MR Egger、Weighted median、IVW、Simple mode、Weighted mode; Beta: risk index; Se: standard error; P: P-value; OR(95%CI):odds ratio(95%confidence interval); Forest plot: Visualize the causal effect of COVID-19 susceptibility and severity on the risk of epigenetic age by five methods(The standard line is the line of "X=0" (red dashed line)), The blue marker dot is a positive result of P<0.05). Epigenetic age is not a dichotomic variable, so the beta is used as a reference indicator. Beta > 0 indicates this effect allele increases the stroke risk. Otherwise, it reduces the stroke risk (Beta < 0)

Supplementary Figure 4. Mendelian randomization effects forest plots for individual SNPs with COVID-19 susceptibility and severity as exposure and epigenetic age as the outcome

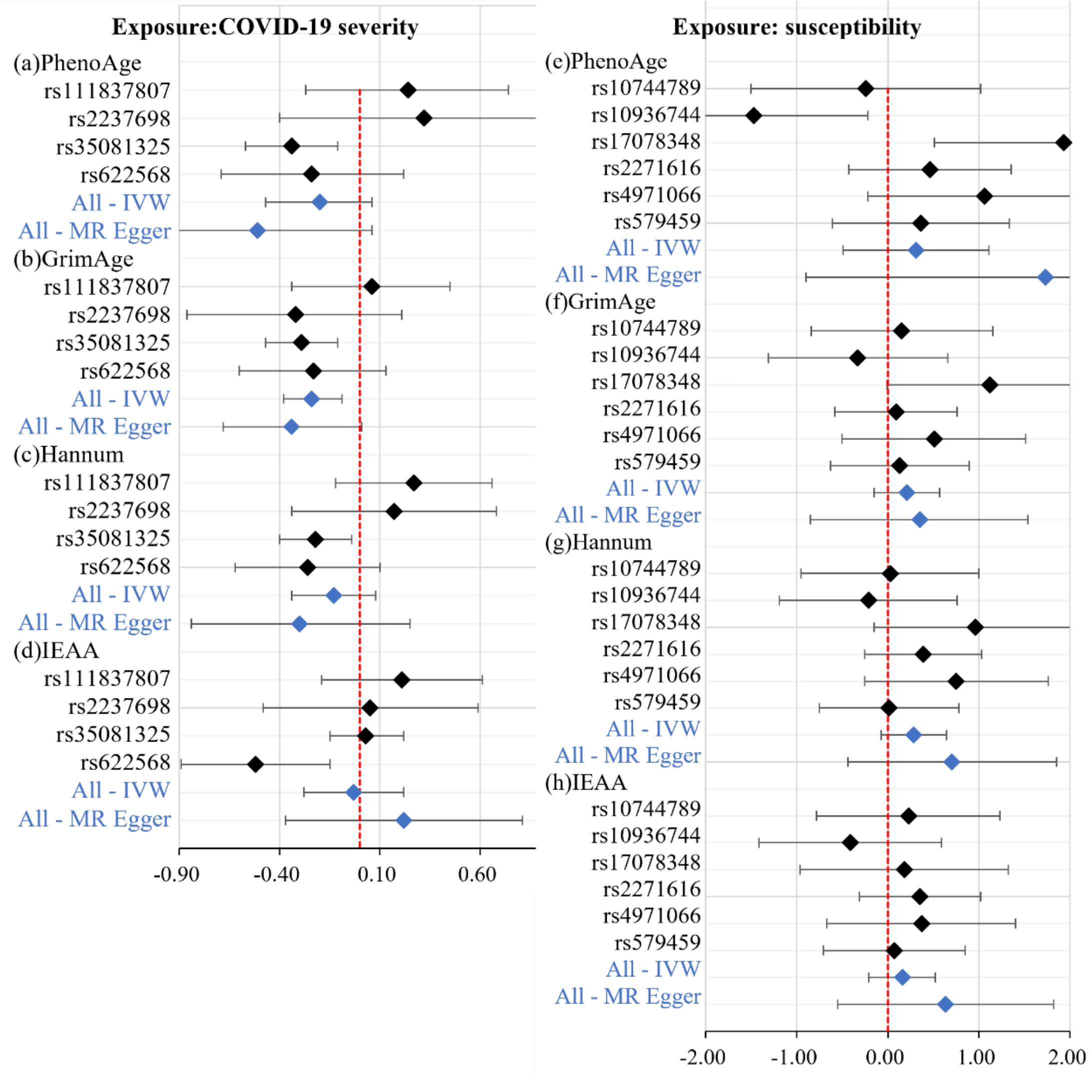

* This figure showed the forest plots for individual SNPs with COVID-19 susceptibility and severity as the exposure, epigenetic age as the outcome. (a)exposure: susceptibility, outcome: PhenoAge; (b)exposure: susceptibility, outcome: GrimAge; (c)exposure: susceptibility, outcome: Hannum; (d)exposure: susceptibility, outcome: HorvathAge; (e)exposure: severity, outcome: PhenoAge; (f)exposure: severity, outcome: GrimAge; (g)exposure: severity, outcome: HannumAge; (h)exposure: severity, outcome: HorvathAge; The blue marker points indicated All-IVW and All-MR Egger

Supplementary Figure 5. OR estimates and 95% CI of the five methods with telomere length as the outcome of exposure, COVID-19 susceptibility and severity

Exposure	Outcome	Method	nSNP	Beta	Se	P	OR(95%CI)
telomere length	severity	MR Egger	48	-0.19	0.35	0.58796	0.83(0.42-1.63)
telomere length	severity	Weighted median	48	-0.31	0.30	0.31311	0.74(0.41-1.33)
telomere length	severity	IVW	48	-0.03	0.20	0.85873	0.97(0.66-1.42)
telomere length	severity	Simple mode	48	-0.52	0.57	0.36465	0.60(0.20-1.81)
telomere length	severity	Weighted mode	48	-0.30	0.29	0.31907	0.74(0.42-1.32)
telomere length	susceptibility	MR Egger	102	0.06	0.09	0.47404	1.07(0.90-1.27)
telomere length	susceptibility	Weighted median	102	0.03	0.08	0.73635	1.03(0.88-1.20)
telomere length	susceptibility	IVW	102	0.00	0.05	0.93182	1.00(0.91-1.11)
telomere length	susceptibility	Simple mode	102	0.13	0.16	0.41020	1.14(0.84-1.56)
telomere length	susceptibility	Weighted mode	102	0.03	0.08	0.75842	1.03(0.87-1.21)

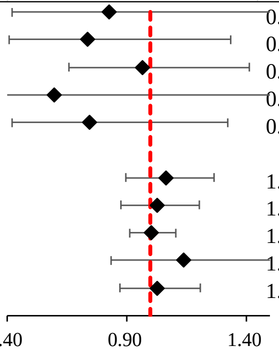

*Methods: MR Egger、Weighted median、IVW、Simple mode、Weighted mode; Beta: risk index; Se: standard error; P: P-value; OR(95%CI):odds ratio(95%confidence interval); Forest plot: Visualize the causal effect of telomere length on the risk of COVID-19 susceptibility and severity by five methods(The standard line is the line of “X=1” (red dashed line))

Supplementary Figure 6. Mendelian randomization effects forest plots for individual SNPs with COVID-19 susceptibility and severity as the exposure and telomere length as the outcome

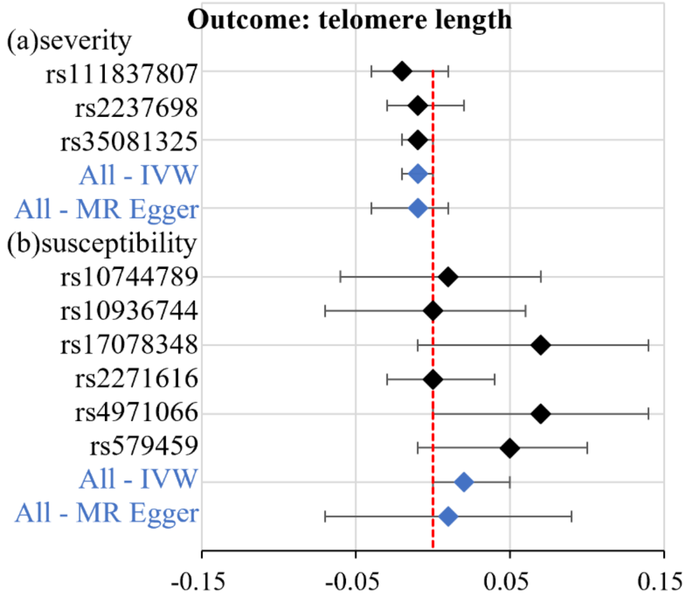

* This figure showed the forest plots for individual SNPs with COVID-19 susceptibility and severity as the exposure, telomere length as the outcome. (a)exposure: severity, outcome: telomere length; (b)exposure: susceptibility, outcome: telomere length; The blue marker points indicated All-IVW and All-MR Egger

Supplementary Figure 7. OR estimates and 95% CI of the five methods with COVID-19 susceptibility and severity as the exposure and telomere length as the outcome

Exposure	Outcome	Method	nSNP	Beta	Se	P	95%CI
severity	telomere length	MR Egger	3	-0.01	0.01	0.49644	(-0.04,0.01)
severity	telomere length	Weighted median	3	-0.01	0.01	0.03857	(-0.02,-0.0006)
severity	telomere length	IVW	3	-0.01	0.01	0.02779	(-0.02,-0.001)
severity	telomere length	Simple mode	3	-0.01	0.01	0.24361	(-0.03,0.002)
severity	telomere length	Weighted mode	3	-0.01	0.01	0.18617	(-0.02,-0.0001)
susceptibility	telomere length	MR Egger	6	0.01	0.04	0.84719	(-0.07,0.09)
susceptibility	telomere length	Weighted median	6	0.01	0.01	0.62696	(-0.02,0.04)
susceptibility	telomere length	IVW	6	0.02	0.01	0.05673	(0.0004,0.05)
susceptibility	telomere length	Simple mode	6	0.01	0.02	0.80644	(-0.04,0.05)
susceptibility	telomere length	Weighted mode	6	0.01	0.02	0.78322	(-0.03,0.04)

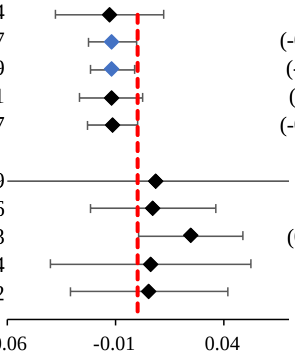

*Methods: MR Egger、Weighted median、IVW、Simple mode、Weighted mode; Beta: risk index; Se: standard error; P: P-value; 95%CI: 95%confidence interval of beta; Forest plot: Visualize causal effect of COVID-19 susceptibility and severity on the risk of telomere length by five methods(The standard line is the line of "X=0" (red dashed line)), The blue marker dot is a positive result of P<0.05). Telomere length is not a dichotomic variable, so the beta is used as a reference indicator.

Supplementary Figure 8. MR leave-one-out sensitivity analysis 'exposure' on 'telomere length'

Exposure	Outcome	SNP	MR leave-one-out sensitivty analysis	Beta	Se	P	OR(95%CI)
severity	telomere length	rs111837807		-0.01	0.01	0.06237	0.99(0.98-1.00)
severity	telomere length	rs2237698		-0.01	0.01	0.02687	0.99(0.98-1.00)
severity	telomere length	rs35081325		-0.01	0.01	0.24316	0.99(0.97-1.01)
severity	telomere length	All		-0.01	0.01	0.02779	0.99(0.98-1.00)
susceptibility	telomere length	rs10744789		0.03	0.01	0.06233	1.03(1.00-1.06)
susceptibility	telomere length	rs10936744		0.03	0.01	0.04195	1.03(1.00-1.06)
susceptibility	telomere length	rs17078348		0.02	0.01	0.10964	1.02(1.00-1.05)
susceptibility	telomere length	rs2271616		0.04	0.01	0.01071	1.04(1.01-1.07)
susceptibility	telomere length	rs4971066		0.02	0.01	0.11974	1.02(1.00-1.04)
susceptibility	telomere length	rs579459		0.02	0.01	0.17027	1.02(0.99-1.05)
susceptibility	telomere length	All		0.02	0.01	0.04673	1.02(1.00-1.05)

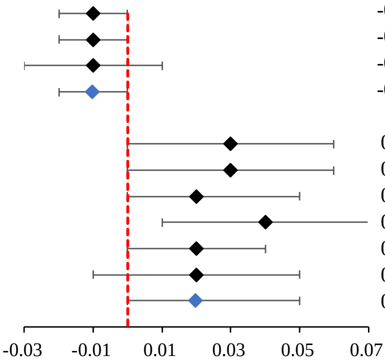

*"Leave-one-out" plot to visualize the causal effect of COVID19 severity and susceptibility on the risk of telomere length when leaving one SNP out.

Supplementary Figure 9. Funnel plots of aging and COVID-19 susceptibility and severity

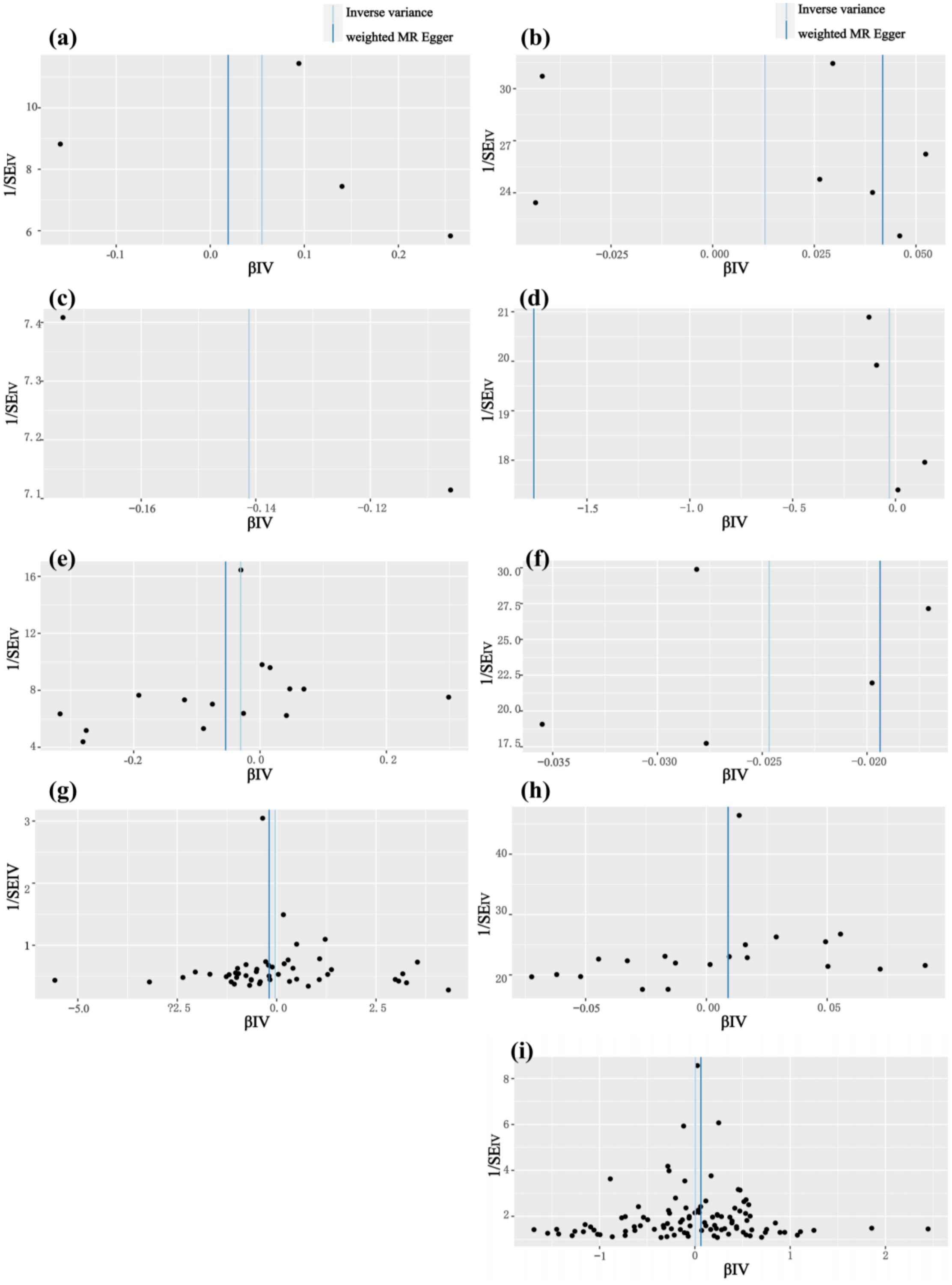

Horizontal ordinate: beta value of the instrumental variables; Vertical coordinates: 1/SE (SE: Standard Error) of the instrumental variables. (a)exposure: PhenoAge, outcome: severity; (b)exposure: PhenoAge, outcome: susceptibility; (c)exposure: GrimAge, outcome: severity; (d)exposure: GrimAge, outcome: susceptibility; (e)exposure: HorvathAge, outcome: severity; (f)exposure: HannumAge, outcome: susceptibility; (g)exposure: telomere length, outcome: severity; (h)exposure: HorvathAge, outcome: susceptibility; (i)exposure: telomere length, outcome: susceptibility.

Supplementary Figure 10. Funnel plots of COVID-19 susceptibility and severity and aging

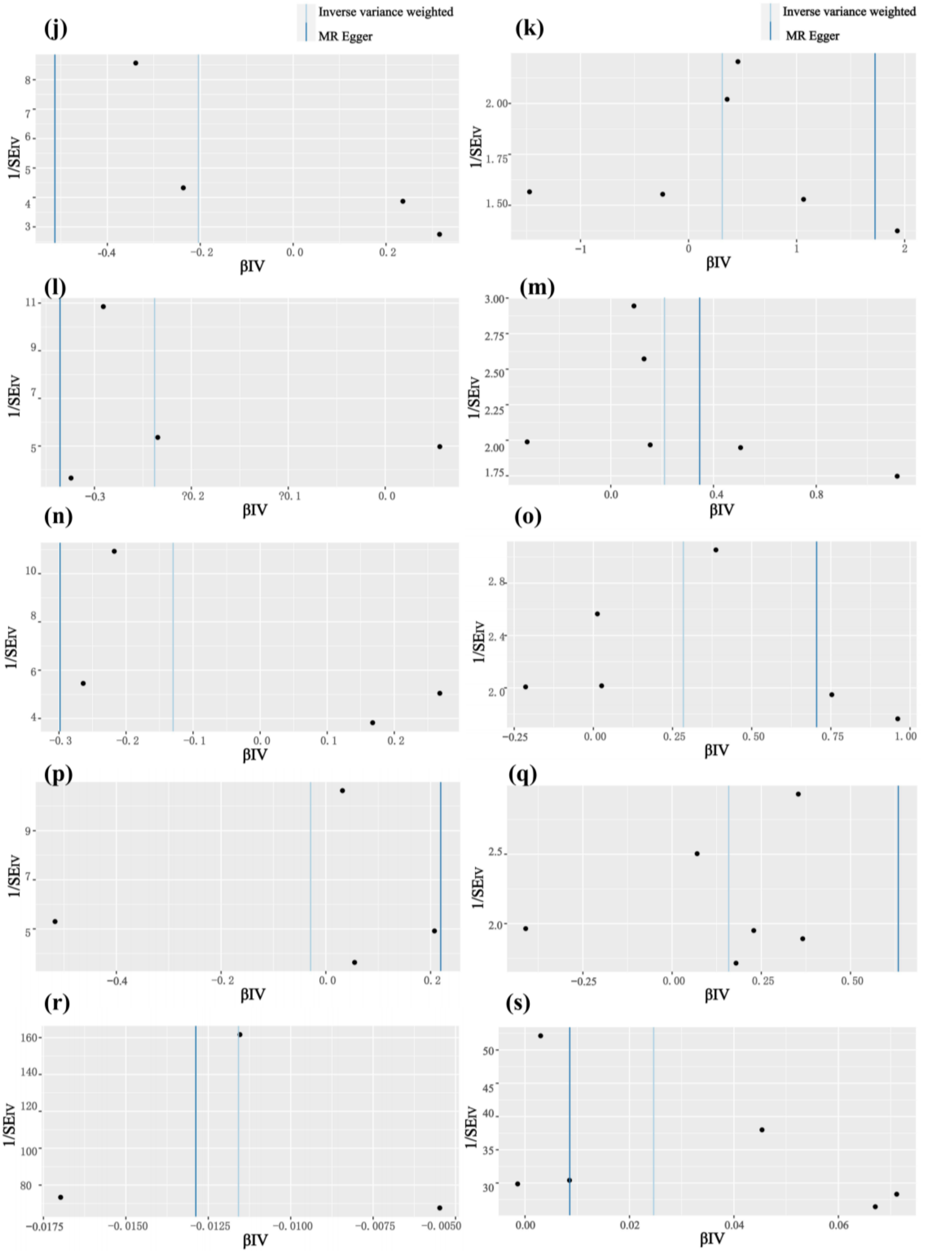

Horizontal ordinate: beta value of the instrumental variables; Vertical coordinates: 1/SE (SE: Standard Error) of the instrumental variables. (j)exposure: severity, outcome: PhenoAge; (k)exposure: susceptibility, outcome: PhenoAge; (l)exposure: severity, outcome: GrimAge; (m)exposure: susceptibility, outcome: GrimAge; (n)exposure: severity, outcome: HannumAge; (o)exposure: susceptibility, outcome: HannumAge; (p)exposure: severity, outcome: HorvathAge; (q)exposure: susceptibility, outcome: HorvathAge; (r)exposure: severity, outcome: telomere length; (s)exposure: susceptibility, outcome: telomere length.
